# Synaptic Plasticity in Cardiac Innervation and Its Potential Role in Atrial Fibrillation

**DOI:** 10.3389/fphys.2018.00240

**Published:** 2018-03-20

**Authors:** Jesse L. Ashton, Rebecca A. B. Burton, Gil Bub, Bruce H. Smaill, Johanna M. Montgomery

**Affiliations:** ^1^Department of Physiology, University of Auckland, Auckland, New Zealand; ^2^Department of Pharmacology, Oxford University, Oxford, United Kingdom; ^3^Department of Physiology, McGill University, Montreal, QC, Canada; ^4^Auckland Bioengineering Institute, University of Auckland, Auckland, New Zealand

**Keywords:** atria, innervation, ganglionated plexi, synapse plasticity, atrial fibrillation, LTP

## Abstract

Synaptic plasticity is defined as the ability of synapses to change their strength of transmission. Plasticity of synaptic connections in the brain is a major focus of neuroscience research, as it is the primary mechanism underpinning learning and memory. Beyond the brain however, plasticity in peripheral neurons is less well understood, particularly in the neurons innervating the heart. The atria receive rich innervation from the autonomic branch of the peripheral nervous system. Sympathetic neurons are clustered in stellate and cervical ganglia alongside the spinal cord and extend fibers to the heart directly innervating the myocardium. These neurons are major drivers of hyperactive sympathetic activity observed in heart disease, ventricular arrhythmias, and sudden cardiac death. Both pre- and postsynaptic changes have been observed to occur at synapses formed by sympathetic ganglion neurons, suggesting that plasticity at sympathetic neuro-cardiac synapses is a major contributor to arrhythmias. Less is known about the plasticity in parasympathetic neurons located in clusters on the heart surface. These neuronal clusters, termed ganglionated plexi, or “little brains,” can independently modulate neural control of the heart and stimulation that enhances their excitability can induce arrhythmia such as atrial fibrillation. The ability of these neurons to alter parasympathetic activity suggests that plasticity may indeed occur at the synapses formed on and by ganglionated plexi neurons. Such changes may not only fine-tune autonomic innervation of the heart, but could also be a source of maladaptive plasticity during atrial fibrillation.

## Introduction

Cardiac arrhythmias are devastating disorders in which normal sinus rhythm is disrupted, resulting in the heart beating too rapidly, slowly, or erratically, thereby impairing cardiac function. The most common cardiac arrhythmia is atrial fibrillation (AF): AF affects 2.5–3.2% of people worldwide, with ~5 million new cases reported annually (Chugh et al., 2014). In AF, atrial electrical activation is rapid and disorganized leading to irregular and often rapid ventricular rhythm. AF disrupts the reservoir and contactile functions of the atria, which impairs ventricular filling and also results in stasis of blood in the left atrium in particular (Staerk et al., 2017). The prevalence of AF increases with aging (Benjamin et al., 1994; Chugh et al., 2014) and it has significant clinical consequences including a 5-fold increase in stroke, a 3-fold increase in heart failure and a doubling of risk for dementia (Benjamin et al., 1994; Chugh et al., 2014).

The hallmark of AF is rapid activation of the atria from one or more localized sources, which can be either focal discharges or self-sustaining circuits of re-entrant activity. Atrial myocardium distal to the arrhythmia source cannot follow the high frequency driver and consequently conduction becomes slow and irregular (Schotten et al., 2011). The progressive nature of this rhythm disturbance is acknowledged in the observation that “AF begets AF” (Wijffels et al., 1995). Repeated episodes of paroxysmal AF, which terminate spontaneously in hours, lead eventually to persistent AF. In persistent AF, atrial electrical and structural remodeling amplifies the electrophysiological instability that drives AF and the re-entrant substrates that sustain it (Iwasaki et al., 2011). It is well established that the autonomic nervous system contributes significantly to this process (Esler, 1992; Chen et al., 2014; Linz et al., 2014; Ardell and Armour, 2016). Sympathovagal discharge is a common trigger for paroxysmal AF (Tan et al., 2008; Chou and Chen, 2009). Specifically, it is thought to be proarrhythmic by enhancing delayed afterdepolarisation related ectopic activity through increasing β-adrenoceptor-dependent diastolic Ca^2+^ leak (Dobrev et al., 2011), and stabilizing re-entrant activity by reducing atrial action potential duration through increased acetylcholine-dependent K+ current (Kneller et al., 2002). Atrial sympathetic hyperinnervation and remodeling of the autonomic nervous system are both contributors to positive feedback loops that promote persistent and recurrent AF (Gould et al., 2006; Tan et al., 2008; Chou and Chen, 2009; Iwasaki et al., 2011). There is evidence of imbalance between sympathetic and parasympathetic components of the autonomic nervous system at both effector and end-organ levels (Chen and Tan, 2007; Czick et al., 2016; Kuyumcu et al., 2017). Furthermore, it is argued that progressive remodeling of the atrial neural plexus in persistent AF contributes to the maintenance of electrical instability (Chen et al., 2010, 2014; Shen et al., 2012). Despite this, we lack detailed knowledge of the structure and function of synapses formed on and by neurons within the atrial neural plexus and how these change with AF.

### Extrinsic and intrinsic innervation of the atria

The atria receive rich innervation from the autonomic branch of the peripheral nervous system (Hillarp, 1960; Skok, 1973; Pardini et al., 1989; Tan et al., 2006; Choi et al., 2010; Chen et al., 2014; Linz et al., 2014). Specifically, the autonomic sympathetic and parasympathetic nervous systems control normal heart rhythm and the heart's susceptibility to atrial and ventricular arrhythmias (Armour, 2008; Choi et al., 2010; Gibbons et al., 2012; Chen et al., 2014; Linz et al., 2014). Sympathetic nerves mediating control of cardiac function originate within the intermediolateral column of the spinal cord and extend to paravertebral ganglia situated from levels C1 to T5, which include the superior cervical ganglia as well as the cervico-thoracic (stellate) ganglia and thoracic ganglia (Kawashima, 2005). Cardiac nerves originating from these ganglia track to the base of the heart along the brachiocephalic trunk, common carotid and subclavian arteries as well as the superior vena cava (Kawashima, 2005). Parasympathetic cardiomotor neurons are situated in medial regions of the medulla oblongata (nucleus ambiguus and dorsal motor nucleus) and issue fibers to the atria via the bilateral vagus nerves (Spyer, 2011).

Most of the sympathetic efferent fibers directly innervate the myocardium or form synapses with neurons in cardiac ganglia located throughout the heart (Armour et al., 1997; Tan et al., 2006; Linz et al., 2014). These synapses consist of presynaptic axonal varicosities invaginated by the postsynaptic cardiomyocyte membrane which contains high densities of adrenergic receptors, adhesion and scaffold proteins (Landis, 1976; Shcherbakova et al., 2007). Hyperactive sympathetic activity is a major feature of heart disease, significantly contributing to the high arrhythmia burden and sudden cardiac death (Chen et al., 2001; Shanks et al., 2013; Ajijola et al., 2015), and recent research has revealed that this is predominantly driven by the postganglionic sympathetic neurons (Larsen et al., 2016a,b). Specifically, hypertension induces increases in membrane calcium currents, intracellular calcium, and cyclic nucleotide signaling in sympathetic stellate neurons, resulting in an increase in noradrenaline release (Shanks et al., 2013; Larsen et al., 2016a,b). Stimulation of sympathetic neurons can redistribute postsynaptic adrenergic receptors on the surface of cardiomyocytes (Shcherbakova et al., 2007). Together these data show that both pre- and post-synaptic changes can readily occur in transmission at sympathetic neuro-cardiac synapses.

**Figure 1:**
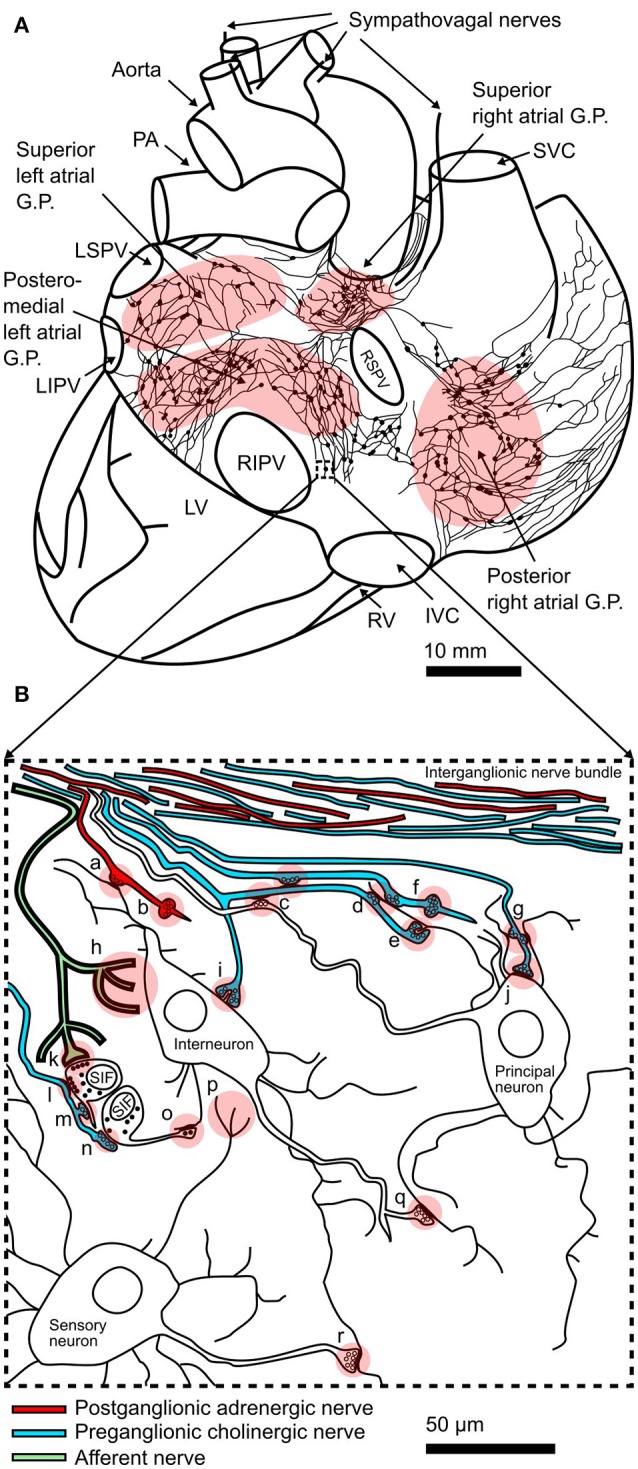
Structure of the intracardiac plexus and synaptic connections within: **(A)** Drawing of a posterior view of the human heart and major vessels showing the locations of posterior atrial ganglionated plexi (GP) and interconnecting nerves. This schematic representation of the plexus is derived from example reconstructions of acetylcholinesterase positive nerves and ganglia on the surface of juvenile atria (Pauza et al., 2000). Sympathovagal nerves enter the heart by coursing down the aorta and superior vena cava (SVC) to innervate the superior atrial GP. The locations of the pulmonary arteries (PA), left superior pulmonary vein (LSPV), left inferior pulmonary vein (LIPV), right superior pulmonary vein (RSPV), right inferior pulmonary vein (RIPV), left ventricle (LV), right ventricle (RV), and inferior vena cava (IVC) are shown. **(B)** Schematic representation of interconnectivity in cardiac ganglia showing types of synapses seen in electron microscopy studies (Shvalev and Sosunov, 1985; Armour et al., 1997; Pauziene and Pauza, 2003): (a) axo-dendritic synapse formed by adrenergic nerve terminal; (b) adrenergic varicosity without glial sheath; (c) axo-axonal synapses; (d) two axons forming axo-dendritic synapses on a single dendrite; (e) axo-dendritic synapse on dendritic spine; (f) cholinergic varicosity without glial sheath; (g) a single axon forming axo-dendritic synapses on two dendrites; (h) afferent nerve ending; (i) axo-dendritic synapse on small spine like protrusion from soma; (j) axo-somatic synapse; (k) contact of afferent nerve terminal with SIF cell; (l) efferent (soma-axonal) synapse made by SIF cell with cholinergic nerve terminal; (m) synapse formed between cholinergic nerve terminal and process of SIF cell; (n) afferent (axo-somatic) synapse made between SIF cell and cholinergic nerve terminal; (o) synapse formed between process of SIF cell and neuronal dendrite; (p) sensory neuron afferent nerve ending; (q) axo-dendritic synapse between neurons within the GP; (r) axo-dendritic synapse between sensory neuron and other neuronal types. Modified from Shvalev and Sosunov (1985).

Parasympathetic fibers form synapses with clusters of cardiac ganglia neurons located on the surface of the heart (Figure 1; Armour, 2008; Linz et al., 2014; Wake and Brack, 2016). These clusters are termed ganglionated plexi (GP), or “little brains” (Armour, 2008), and they are proposed to act as local coordinators of cardiac electrical and mechanical properties (Horackova and Armour, 1995; Choi et al., 2010; Linz et al., 2014; Ardell and Armour, 2016). In humans, approximately 14,000 GP neurons are located on the heart surface, with many clustered around the pulmonary veins (Armour et al., 1997). Increasing evidence supports the hypothesis that GP neurons can independently modulate neural control of the heart (Horackova and Armour, 1995; Arora et al., 2003; Heaton et al., 2007; Choi et al., 2010; Gibbons et al., 2012; Chen et al., 2014; Linz et al., 2014). For example, GP neurons are proposed to play a critical role in the development and propagation of arrhythmias such as AF (Choi et al., 2010; Gibbons et al., 2012; Chen et al., 2014; Linz et al., 2014), and AF can be induced by direct stimulation of GP sites (Lim et al., 2011; Gibbons et al., 2012). In addition, changes in parasympathetic tone (which increase the risk of arrhythmias, heart failure and mortality), have been proposed to occur in GP (Bibevski and Dunlap, 1999; Arora et al., 2003; Heaton et al., 2007).

## Ganglionated plexi

### GP structure and neuron function

Although initially defined as clusters of cholinergic neurons, GP neurons show significant heterogeneity in their morphology, chemical composition, and physiology (Edwards et al., 1995; Horackova et al., 1999; Richardson et al., 2003; Rimmer and Harper, 2006; Tan et al., 2006; McAllen et al., 2011; Wake and Brack, 2016). Immunocytochemical analysis has revealed multiple neurochemical subtypes of GP neurons: while choline acetyltransferase (ChAT) is expressed in all principal neurons, subpopulations express other transmitters and neuropeptides including nitric oxide, serotonin, and neuropeptide Y (Mawe et al., 1996; Horackova et al., 1999; Singh et al., 1999; Richardson et al., 2003; Adams and Cuevas, 2004; Wake and Brack, 2016). Small clusters of catecholaminergic neurons, termed SIF (small intensely fluorescent) cells, constitute 5% of GP neurons (Horackova et al., 1999; Slavíková et al., 2003) where they modulate synaptic transmission of the cholinergic neurons (McGrattan et al., 1987; Gagliardi et al., 1988; Adams and Cuevas, 2004). The presence of multiple neurochemical variants suggests differential roles for peptides and neurotransmitters in modulating GP neuron function. Distinct subtypes of neurons within GP have also been defined electrophysiologically based on action potential kinetics, ability to fire bursts of action potentials, rectification properties and synaptic input (Edwards et al., 1995; McAllen et al., 2011). This heterogeneity within GP suggests the neurons play different roles in controlling electrical signals to the heart (Ardell and Armour, 2016). Moreover, the ability of GP neurons to modulate the level of parasympathetic activity to the heart also suggests that synaptic communication from GP neurons can be altered. These synaptic changes may not only fine-tune autonomic activation of the heart, but could also likely be a source of maladaptive changes including arrhythmogenesis (Choi et al., 2010; Gibbons et al., 2012; Chen et al., 2014; Ajijola et al., 2015; Ardell et al., 2016).

### Clinical importance of GP neurons

Clinically, AF is treated pharmacologically with rate and rhythm controllers (Lafuente-Lafuente et al., 2015; Hanley et al., 2016; which have variable efficacy and trigger ventricular arrhythmias), and by the pulmonary vein isolation procedure. In this technique, an interior ring or “firebreak” is created within each pulmonary vein to stop aberrant electrical impulses that can trigger AF from reaching the heart (Haissaguerre et al., 1998; Lancaster et al., 2016). This procedure has been most effective in reversing paroxysmal AF compared with persistent AF, however initial success rates at 12 months drop significantly beyond 2 years (Kron et al., 2010; Weerasooriya et al., 2011; Calkins et al., 2012; Zheng et al., 2013). Ablation of the GP sites has been combined with pulmonary vein isolation, and these two procedures appear to increase the numbers of patients free of AF, however results are variable, and long-term efficacy is unknown (Scherlag et al., 2006; Pokushalov et al., 2009; Kron et al., 2010; Katritsis et al., 2011; Calkins et al., 2012; Zheng et al., 2013). Moreover, GP neurons also innervate the ventricles and modulate ventricular function, raising concern of increased susceptibility to ventricular arrhythmias after ablation procedures (Pappone et al., 2004; Osman et al., 2010; Buckley et al., 2016; Jungen et al., 2017). It is critical that in order to advance GP ablation techniques and increase their reproducibility and success rates that we gain a detailed understanding of the physiological properties of the GP neurons in the aged or arrhythmic states, and how changes in their function may trigger and drive AF.

## Beyond the brain – does synaptic plasticity occur in neurons innervating the heart?

“Plasticity” is defined as the ability of neurons to alter their strength of communication at synapses (Bliss and Lomo, 1973; Dudek and Bear, 1992; Genoux and Montgomery, 2007; Nabavi et al., 2014). Synapse plasticity is a critical process in the brain, and a major area of neuroscience research as it has been shown to underlie learning and memory, as well as changes in sensory and motor functions (Genoux and Montgomery, 2007; Huang et al., 2007; Lee et al., 2014; Nabavi et al., 2014; Leighton and Lohmann, 2016). High-frequency stimulation paradigms induce increases in synaptic efficacy that last for seconds (i.e., short-term plasticity; Dobrunz et al., 1997; Jackman and Regehr, 2017), or from minutes to hours or days, referred to as long-term potentiation (LTP; Bliss and Lomo, 1973). Alternatively, low frequency stimulation paradigms induce long term depression (LTD) of synaptic efficacy (Dudek and Bear, 1992). The *induction* of LTP and LTD is dependent on activation of NMDA-type glutamate receptors (Harris et al., 1984; Morris et al., 1986; Dudek and Bear, 1992). The mechanisms underpinning the *expression* of these changes in synaptic strength vary between brain regions. Specifically, LTP/LTD paradigms can induce changes in postsynaptic receptor surface number, conductance, and distribution, the probability of presynaptic transmitter release, and/or ultrastructural changes in synaptic protein localisation (Hayashi et al., 2000; Montgomery et al., 2001; Castillo et al., 2002; Mellor et al., 2002; Ehlers et al., 2007; Volk et al., 2015; Tang et al., 2016). However, in contrast to the brain, less is known about the mechanisms of short and long-term synaptic plasticity in the neurons that innervate the heart.

### Synaptic plasticity in cardiac sympathetic ganglia

Both short and long-term plasticity mechanisms have been described in the peripheral synapses within sympathetic ganglia. In the stellate and superior cervical sympathetic ganglia that innervate the heart, short-term increases in the strength of synaptic transmission occur in response to a single action potential or a short train of impulses (Bennett et al., 1976; Lin et al., 1998), and longer bursts of high frequency stimulation of the preganglionic nerve result in enhancement of postsynaptic responses and heart rate (Alonso-deFlorida et al., 1991; Bachoo and Polosa, 1991; Aileru et al., 2004). Conversely, low frequency stimulation can induce LTD (Alkadhi et al., 2008). Induction of ganglionic LTP and LTD (gLTP/gLTD) is not dependent on transmission via nicotinic, adrenergic, muscarinic or adenosine receptors, but requires activation of 5-HT_3_ receptors by serotonin, potentially released from SIF cells (Alkadhi et al., 1996, 2008). Both pre- and postsynaptic expression mechanisms have been implicated in gLTP (Alkadhi et al., 2005), with increases in evoked acetylcholine (ACh) release (Briggs et al., 1985) and postsynaptic sensitivity to ACh observed (Bachoo and Polosa, 1991). More recently, both pre- and postsynaptic intracellular calcium changes have been shown to contribute equally to gLTP (Vargas et al., 2011), and the potential involvement of nitric oxide signaling (Altememi and Alkadhi, 1999) supports a trans-synaptic form of gLTP (Vargas et al., 2011) that can be enhanced by neurotrophins to regulate sympathetic tone (Arias et al., 2014).

The mechanisms underpinning gLTP likely contribute to the enhanced sympathetic drive seen in conditions associated with heart disease and AF (Alkadhi and Alzoubi, 2007). In spontaneously hypertensive rats (SHRs), synaptic transmission is augmented as shown by increased ACh release (Magee and Schofield, 1992, 1994), greater recruitment of postganglionic neurons (Magee and Schofield, 1992), and faster spike frequency adaptation in SHR ganglia (Yarowsky and Weinreich, 1985). Increased sympathetic stimulation may increase presynaptic activity to induce gLTP observed *in vivo* in sympathetic ganglia in SHRs (Alzoubi et al., 2010). Additional evidence of gLTP *in vivo* is the inhibition of baseline ganglionic transmission by 5-HT_3_ receptor antagonists in sympathetic ganglia from SHRs but not age-matched controls (Alkadhi et al., 2001). Further gLTP cannot be induced, indicating occlusion of the plasticity mechanism (Alkadhi and Alzoubi, 2007). Chronic treatment with 5-HT_3_ receptor antagonists also reduces blood pressure in SHRs (Alkadhi et al., 2001) but its effect on atrial arrhythmia burden in this model is unknown.

### Synaptic plasticity in the intracardiac plexus

Within GP, the cholinergic and catecholaminergic neurons possess large numbers of asymmetrical axodendritic synapses and project axons to neurons within the same or different ganglion (Figure 1; Armour et al., 1997; Klemm et al., 1997; Horackova et al., 1999; Richardson et al., 2003; Tan et al., 2006; Armour, 2008), suggesting that significant synaptic communication occurs between networks of GP neurons. Electrophysiological recordings from synapses within the intracardiac plexus are hampered by difficulty accessing GP neurons given their proximity to the heart and great vessels, and the extensive connective tissue surrounding the ganglia. Intracellular recordings from GP neurons have been performed in the working heart-brainstem preparation (McAllen et al., 2011). These recordings revealed the presence of subthreshold synaptic potentials and silent synapses, indicating that significant capacity exists for increasing synaptic strength within GP, which could alter and/or restore vagal tone (McAllen et al., 2011). Multiple studies have measured changes in postsynaptic neuronal excitability as an indication of synaptic efficacy in the intracardiac plexus following chronic spinal cord stimulation or myocardial infarction, suggesting altered neurotransmission in the intracardiac plexus contributes to altered parasympathetic control of the heart (Bibevski and Dunlap, 1999; Ardell et al., 2014; Hardwick et al., 2014; Rajendran et al., 2016; Smith et al., 2016). After myocardial infarction, the observed overall reduction in network connectivity (Rajendran et al., 2016) suggests depression of synaptic transmission (i.e., LTD) within the intracardiac nervous system, however this may differ at afferent versus efferent synaptic inputs. With regards to AF, enhanced interaction at the level of the GP network through changes in local circuit neuron function have been proposed to be a factor contributing to AF substrate (Beaumont et al., 2013; Ardell et al., 2016). Possible factors altering ganglionic neurotransmission include changes in postsynaptic nicotinic receptor expression (Bibevski and Dunlap, 2011) and dysfunctional NO-cGMP signaling in postganglionic neurons (Heaton et al., 2007). Intriguingly, NMDA receptors are abundantly expressed in the atrium, including in the GP (Gill et al., 2007), and their activation is associated with increased arrhythmogenesis, AF inducibility, and atrial fibrosis (Shi et al., 2014, 2017). NMDA receptors are critical for the induction of synaptic plasticity in the brain, suggesting that NMDA receptors in the heart also play a role in inducing plasticity within GP, and contribute to autonomic dysfunction in arrhythmias such as AF.

Significant evidence indicates the neuropeptide pituitary adenylate cyclase-activating polypeptide (PACAP) is involved in plasticity at GP synapses. PACAP is localized to parasympathetic preganglionic fibers (Calupca et al., 2000; Richardson et al., 2003) and GP neurons express PAC_1_ receptors (Braas et al., 1998). PACAP modulates nicotinic neurotransmission in the ciliary ganglion by enhancing presynaptic quantal ACh release via trans-synaptic action of NO (Pugh et al., 2010; Jayakar et al., 2014). Postsynaptically, PACAP increases the agonist affinity of GP nicotinic receptors through G-protein signaling (Liu et al., 2000). High frequency stimulation of nerve bundles within the intracardiac plexus results in a slow postsynaptic depolarisation and a sustained increase in excitability of GP neurons that is thought to be at least partially mediated by PACAP (Tompkins et al., 2007). This increase in excitability is driven by enhanced current through hyperpolarization-induced nonselective cationic (I_h_; Tompkins et al., 2009) and T/R-type calcium channels (Tompkins et al., 2015). The enhanced excitability of GP neurons likely contributes to the PACAP induced AF seen in dogs (Hirose et al., 1997) and guinea pigs (Chang et al., 2005).

## Future directions

The detailed knowledge of plasticity mechanisms in the brain has resulted from precise imaging and electrophysiological analysis of synaptic properties (e.g., Hayashi et al., 2000; Montgomery et al., 2001; Ehlers et al., 2007; Fourie et al., 2014; Tang et al., 2016). To gain comparable knowledge of short and long-term plasticity mechanisms in the innervation of the heart, similar high-resolution techniques need to be applied to synapses formed on and by cardiac parasympathetic and sympathetic neurons, especially in human tissue where some aspects of GP circuitry and cell composition appear to differ (Armour et al., 1997; Pauziene and Pauza, 2003; Hoover et al., 2009). Animal models of AF are also important, as recording changes in GP neuron and synapse function during and after the onset of arrhythmia will provide evidence of whether plasticity does occur at GP synapses with changes in heart rhythm. In more intact systems, such as the working heart-brainstem and the innervated heart preparations (Brack et al., 2004; Ng et al., 2007; McAllen et al., 2011; Ashton et al., 2013), this would enable researchers to determine whether changes in synaptic strength can increase or decrease autonomic tone to the heart, and play a major role in generating the aberrant electrical impulses in the GP around the pulmonary veins that can trigger and drive AF.

## Author contributions

JM and JA initiated the review topic and designed the review. All authors contributed to the writing, editing, and approval of the manuscript.

### Conflict of interest statement

The authors declare that the research was conducted in the absence of any commercial or financial relationships that could be construed as a potential conflict of interest.
